# Expression Pattern of *nos1* in the Developing Nervous System of Ray-Finned Fish

**DOI:** 10.3390/genes13050918

**Published:** 2022-05-20

**Authors:** Giovanni Annona, José Luis Ferran, Pasquale De Luca, Ivan Conte, John H. Postlethwait, Salvatore D’Aniello

**Affiliations:** 1 Biology and Evolution of Marine Organisms (BEOM), Stazione Zoologica Anton Dohrn, 80121 Napoli, Italy; 2 Research Infrastructure for Marine Biological Resources Department (RIMAR), Stazione Zoologica Anton Dohrn, 80121 Napoli, Italy; p.deluca@szn.it; 3 Department of Human Anatomy and Psychobiology, Faculty of Medicine, University of Murcia, 30120 Murcia, Spain; jlferran@um.es; 4 Institute of Biomedical Research of Murcia—IMIB, Virgen de la Arrixaca University Hospital, 30120 Murcia, Spain; 5 Telethon Institute of Genetics and Medicine, 80078 Pozzuoli, Italy; ivan.conte@unina.it; 6 Department of Biology, University of Napoli Federico II, 80126 Napoli, Italy; 7 Institute of Neuroscience, University of Oregon, Eugene, OR 97401, USA; jpostle@uoregon.edu

**Keywords:** fish, brain evolution, nitric oxide synthase, nNos, neuronal *nos*

## Abstract

Fish have colonized nearly all aquatic niches, making them an invaluable resource to understand vertebrate adaptation and gene family evolution, including the evolution of complex neural networks and modulatory neurotransmitter pathways. Among ancient regulatory molecules, the gaseous messenger nitric oxide (NO) is involved in a wide range of biological processes. Because of its short half-life, the modulatory capability of NO is strictly related to the local activity of nitric oxide synthases (Nos), enzymes that synthesize NO from L-arginine, making the localization of *Nos* mRNAs a reliable indirect proxy for the location of NO action domains, targets, and effectors. Within the diversified actinopterygian *nos* paralogs, *nos1* (alias *nnos*) is ubiquitously present as a single copy gene across the gnathostome lineage, making it an ideal candidate for comparative studies. To investigate variations in the NO system across ray-finned fish phylogeny, we compared *nos1* expression patterns during the development of two well-established experimental teleosts (zebrafish and medaka) with an early branching holostean (spotted gar), an important evolutionary bridge between teleosts and tetrapods. Data reported here highlight both conserved expression domains and species-specific *nos1* territories, confirming the ancestry of this signaling system and expanding the number of biological processes implicated in NO activities.

## 1. Introduction

Ray-finned fishes (*Actinopterygii*) experienced the largest radiation within vertebrates [1,2,3], colonizing almost all aquatic habitats. Within ray-finned fishes, the crown group Neopterygii includes holosteans and teleosts, which evolved several morphological and functional adaptations that facilitated their expansion, including complex neural networks and innovative modulatory neurotransmitter pathways that contributed to their evolutionary success. Nitric oxide (NO), an ancient gaseous regulatory molecule [4], is involved in a wide range of biological processes, including the elaboration of olfactory, visual, and neuroendocrine stimuli [5], learning and memory [6], and the modulation of social behaviors [7]. Tetrapods have three paralogs encoding the enzymes that produce NO, the nitric oxide synthase (*Nos*) genes *Nos1*, *Nos2,* and *Nos3*. These three paralogs appear to have arisen in the vertebrate genome duplication events [8]. *Nos1* and *Nos3* are constitutively expressed and *Nos2* is induced by pro-inflammatory processes [9]. Compared to tetrapods, the fish *nos* family shows a more complex evolutionary scenario due to several gene duplications and gene loss events [10]. *Nos1,* in contrast to the other two paralogs, is present ubiquitously across the gnathostome lineage [8,10,11]. Nos1 is the predominant source of NO in brain neurons [11], and since this gaseous neurotransmitter cannot be stored within cells, its functional properties depend on continuous new synthesis [12]. 

A discrete number of brain areas expressing *nos* have been mapped in adult teleosts, including preoptic area, hypothalamus, optic tectum, rhombencephalic neurons, pituitary cells, olfactory bulb, thalamus, hypothalamus, tegmentum and cerebellum, cranial nerves, and spinal cord nuclei [5,13,14,15,16,17,18,19,20]. Moreover, a recent study on the expression pattern of *nos1* in the adult brain of spotted gar (*Lepisosteus oculatus*), a representative of the holosteans, the sister group to the teleosts, described the pattern of nitrergic neuronal system highlighting shared and derived traits in respect to teleosts [21]. Nitrergic neurons have been characterized during fish embryogenesis exclusively in cyprinids, where they play key roles in neurogenesis, organogenesis, and early brain physiology [22,23,24,25]. In this work, to help define regions of the brain in which the nitrergic system might be essential for normal brain development and function, we used a transcriptional approach as the first choice in understanding *nos1* action. We investigated the conservation of *nos1* gene expression domains in embryos and larvae of two teleosts (zebrafish (*Danio rerio*) and medaka (*Oryzias latipes*)) and an early-branching holostean spotted gar (*L. oculatus)*, which has a better preserved ancestral genome organization than teleosts and thus provides an important evolutionary bridge between teleosts and tetrapods in cross-species comparisons [26,27]. The characterization of anatomical derivatives of the central nervous system among evolutionarily distant fish species can be facilitated by the comparison of the specific location of gene expression patterns within the neuronal Bauplan [28].

The investigation of potential NO sources in fish clades indicated above furthers our understanding of the functional evolution of this ancient signaling system. This study represents the first comparative analysis of *nos1* expression patterns in development among divergent fish species and suggests specific homologies among nervous systems that pave the way for future detailed comparative work on the functions of NO in developing fish central nervous systems.

## 2. Materials and Methods

### 2.1. Embryo Collections 

Embryos of the three species were fixed in 4% paraformaldehyde (PFA) in 1X phosphate buffered saline with 0.1% Tween20 (PBT) overnight at 4 °C, then dehydrated in increasing concentrations of methanol in 1X PBT until 100% methanol and kept at −20 °C until use. 

Wildtype adult spotted gar (*L. oculatus*) were collected from the Atchafalaya River basin, Louisiana (USA) and cultured in a 2 m diameter tank containing artificial spawning substrate. Spawning was induced by injection of Ovaprim© (0.5 mL/kg). Embryos were raised in fish water (1 ppt salinity) at 24 °C in a 14/10 h light/dark cycle [29]. Developmental stages were annotated by both time in hours or days post-fertilization (hpf or dpf) and by morphological criteria as previously described [30]. 

Zebrafish (*D. rerio*) AB strain broodstock was maintained according to standard methods [31,32]. Zebrafish embryos were obtained by natural spawning, raised at 28 °C, and staged according to time and morphological criteria [33]. 

Medaka (*O. latipes*, Cab Strain) were kept at the Medaka Fish Facility, TIGEM (Telethon Institute of Genetics and Medicine, Pozzuoli, Italy), in standard conditions and staged as described previously [34].

### 2.2. Cloning and Probe Preparation

Total RNA was isolated by the phenol–chloroform method with EUROzol (EuroClone, Pero, Italy) from zebrafish and medaka embryos and from juvenile spotted gar brain. The cDNA was synthesized from 1 µg of total RNA using the SMART PCR cDNA Synthesis Kit (Takara, Kasatsu, Shiga, Japan). Specific primers for *nos1* amplification were designed using zebrafish (NM_131660), medaka (NM_001104855), and spotted gar (XM_015366414) sequences, and are listed in Table S1. Amplicons were cloned into the pGEM-T Easy Vector (Promega, Madison, WI, USA). Specific antisense Digoxygenin-UTP riboprobes were designed to avoid cross-hybridization among *nos* gene paralogs and were synthesized with SP6 or T7 RNA polymerases using the DIG RNA Labeling Kit (Roche, Basel, Switzerland).

### 2.3. In Situ Hybridization

Although in situ hybridization protocols differed slightly among the three species, we tried to use comparable the staining and imaging experimental settings to avoid technical bias and, while we recognize that making quantitative comparisons of in situ hybridization data across genes and species has pitfalls, comparisons of different expression domains within a single individual is internally controlled.

Whole mount in situ hybridization experiments in zebrafish embryos followed the previously described protocol [32] with minor modifications: we removed endogenous melanin pigmentation using bleaching solution ((3% hydrogen peroxide (H_2_O_2_) and 1% potassium hydroxide (KOH) in distilled water (ddH_2_O)) for a few minutes, hybridized at 65 °C, and used BM-Purple as the staining reaction substrate (Roche, Basel, Switzerland). Embryos were washed several times in 1X PBT, mounted in 80% glycerol in PBS, and photographed.

Whole mount in situ hybridization experiments in medaka embryos were carried out as described [35], and after washes and photography were embedded in a mix of bovine serum albumin and gelatin from porcine skin (Sigma-Aldrich, St. Louis, MO, USA), then sectioned using a vibratome at a thickness of 20 µm. Because of skin thickness and weak probe penetration, spotted gar in situ experiments were performed on cryosections following the original protocol described by [36], including modifications reported by [37]. To block the staining reaction, slides were washed in 2X PBT and then mounted as follows: two washes in ddH_2_O, quick dehydration steps through graded ethanol to 100%, clearing in xylene, and coverslipping using Permount (Fisher Scientific, Waltham, MA, USA).

Microscopy was performed using an Axio Imager Z2 with Apotome 2 microscope (Carl Zeiss, Jena, Germany), equipped with an Axiocam 503 color digital camera and Axio Vision software for analysis.

## 3. Results

### 3.1. Expression Pattern of nos1 in the Brain of Zebrafish Embryos and Larvae 

**Figure 1:**
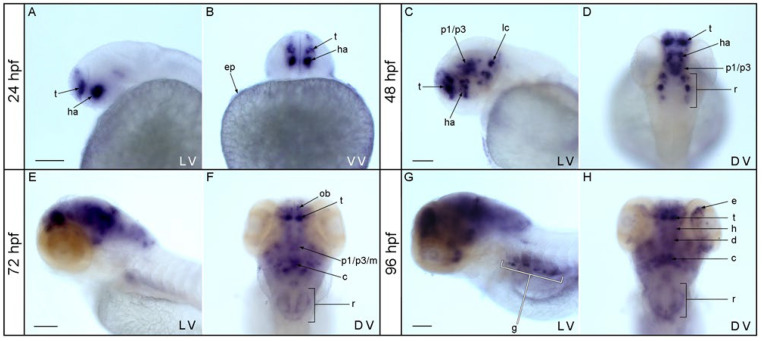
Expression of *nos1* during zebrafish development. At 24 hpf, *nos1* expression appeared in pallial and subpallial telencephalic territories, in the alar hypothalamus, and in epidermis (**A**,**B**). At 48 hpf, *nos1* signal emerged in the telencephalon, in the dorsal portion of the hypothalamus, the basal plate of prosomeres 1 to 3, and in the midbrain and rhombencephalon (**C**,**D**). At 72 hpf, the olfactory bulbs, the pallium in the telencephalon, the basal plate of prosomeres p1-p3, and the midbrain, cerebellum and rhombencephalon were positively marked (**E**,**F**). At 96 hpf, *nos1* transcripts were detected in the telencephalon, hypothalamus, diencephalon proper, midbrain, cerebellum, rhombencephalon, eye, and gut (**G**,**H**). Abbreviations: ah, alar hypothalamus; c, cerebellum; d, diencephalon; e, eye; g, gut; h, hypothalamus; ha, alar hypothalamus; p1/p3/m, basal plate of prosomere 1 to 3 and midbrain; ep, epidermis; lc, locus coeruleus; ob, olfactory bulbs; r, rhombencephalon; t, telencephalon. DV dorsal view; VV, ventral view; LV, lateral view. Scale bar: 500 µm.

To identify *nos1* expression domains in teleosts, we began with zebrafish. Whole mount in situ hybridization experiments were performed on zebrafish embryos at 24 and 48 hpf and on larvae at 72 and 96 hpf. Results showed an increase of *nos1*-positive territories during brain development, confirming data in the literature [24,25,38]. At 24 hpf, *nos1* expression appeared in the pallial and subpallial territories of the telencephalon and the alar hypothalamic region (dorsal portion of hypothalamus) and in the epidermis (Figure 1A,B). At 48 hpf, *nos1* expressing cells appeared in the telencephalon and in the dorsal portion of the hypothalamus as at 24 hpf. Expression of *nos1* was also detected in the diencephalon proper (mainly in the basal plate of prosomere 1, 2, and 3), midbrain basal plate, and various spots in the rhombencephalon (including the locus coeruleus) (Figure 1C,D). At 72 hpf, olfactory bulbs, the pallial and subpallial territories of the telencephalon, and the alar and basal (mainly the retrotuberal) domains of the hypothalamus expressed *nos1*. Moreover, expression also appeared in basal plate derivatives from prosomeres p1 to p3, in the mesencephalon and cerebellum, and in the rhombencephalon (Figure 1E,F). At 96 hpf (Figure 1G,H), *nos1* signal spread to other derivatives in the main areas of the brain mentioned above, but novel signals emerged in several areas of the retina (Figure 1H) and the gut (Figure 1G). Single-cell transcriptomics (scRNA-seq) provides an independent dataset that validates expression domains observed in in situ hybridization experiments. The scRNA-seq data analysis [39] showed that the highest levels of *nos1* expression in zebrafish embryos at 24 and 48 hpf and at 5 dpf is in the periderm and a specific group of midbrain and hindbrain neurons (Figure S1). At mid-value levels of expression, *nos1* was expressed in blood vessels and ionocytes, and at lower levels in liver, muscle, basal skin cells, gill, retina, and in the CNS in the forebrain, floorplate of the midbrain, and in spinal cord progenitor cells, all as confirmed by the in situ hybridization results.

### 3.2. Expression Pattern of nos1 in the Brain of Medaka Embryos and Larvae

**Figure 2:**
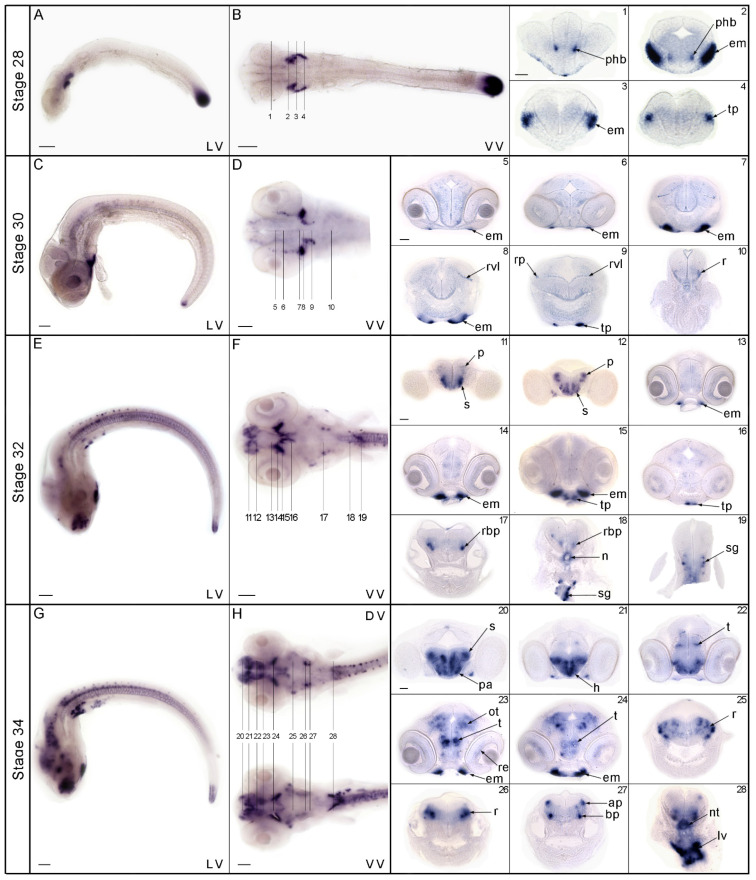
Expression of *nos1* in medaka embryos and larvae. At stage 28, whole-mount in situ hybridization experiments displayed positive cells in the rostral portion of the nervous system and in the tailbud (**A**,**B**). At this stage, sections showed signal around the diencephalon-hypothalamus boundary (1–2), in the extraocular muscle (2–3), and caudally in the thyroid primordium (4). At stage 30 (**C**,**D**), expression was retained in the extraocular muscle and thyroid primordium, (5 to 9) and *nos1*-positive cells were detected in ventricular layer cells of the rhombencephalon (8–9), between the alar and basal plate (9), and in the rhombencephalic area (10). At stage 32 (**E**,**F**), *nos1* was expressed in the subpallial and pallial region (11–12), in the extraocular muscle (13–15), and the thyroid primordium (15–16). Caudally, *nos1* signal was present in the rhombencephalon (17–18), notochord, and sympathetic ganglia (18–19). At stage 34 (**G**,**H**), hybridization signal was present in the subpallium, preoptic area, hypothalamic domains, thalamus, optic tectum, eye and extraocular muscle (21 to 25), basal and alar plate of the rhombencephalon (26 to 28), notochord, and liver primordium (28). Abbreviations: ap, alar plate; bp, basal plate; phb, prethalamus-hypothalamus boundary; em, extraocular muscles: tp, thyroid primordium; h, hypothalamus; lv, liver; n, notochord; ot, optic tectum; pn, pallium nuclei; r, rhombencephalon; rbp, rhombencephalic basal plate; re, retina; rp, rhombencephalic plate; rvl, rhombencephalic ventricular layer; s, subpallium; sg, sympathetic ganglia; t, thalamus; tp, thyroid primordium. DV dorsal view; VV, ventral view; LV lateral view. Scale bar: whole-mount 100 µm; sections 250 µm.

In medaka embryos, in situ hybridization experiments revealed *nos1* expression at stages 28, 30, 32, and 34, according to [34]. Coronal/transverse sections at stage 28 (Figure 2A,B) revealed *nos1* expression in the prosencephalic region of the central nervous system, around the boundary between prethalamus and hypothalamus (Figure 2 (sections 1–2)), and outside the central nervous system (CNS) in a territory that is likely the primordium of extraocular muscles (Figure 2 (sections 2–3)). Caudally, *nos1* was expressed in a group of cells that appear to correspond to the thyroid primordium (Figure 2 (section 4)). At stage 30 (Figure 2C,D), the primordium of extraocular muscles (Figure 2 (section 5–8)) and the putative primordium of the thyroid (Figure 2 (section 9)) continued to be positive. Furthermore, an additional punctate signal appeared in ventricular layer cells of the rhombencephalon close to the fourth ventricle (Figure 2 (sections 8–9)), flanked by superficial post-mitotic cells in the boundary between the alar and basal plate (Figure 2 (section 9)), and in the rhombencephalon (Figure 2 (section 10)). At stage 32 (Figure 2E,F), a new *nos1* expression domain appeared in some of the derivatives of the pallial and subpallial regions of the telencephalon (Figure 2 (section 11–12)). This developmental phase maintained a signal in extraocular muscles (Figure 2 (section 13–15)) and in the postulated thyroid primordium (Figure 2 (sections 15–16)), which at this stage had migrated to the anterior-midline of the body close to the mesencephalon (see stage 30). More posteriorly, *nos1* was expressed in the basal plate of the rhombencephalon (Figure 2 (section 17–18)), around the notochord, and in a few isolated neurons of the sympathetic ganglia (Figure 2 (section 18–19)). At stage 34 (Figure 2G,H), *nos1* signal increased conspicuously in all brain regions, including the forebrain mainly in the subpallium, in the preoptic area, hypothalamic domains (probably in the retrotuberal region), in the thalamus, in some parts of the alar and basal plate of the midbrain, in some domains of the hindbrain, and in extraocular muscle. Additionally, *nos1* signal became detectable in the retina of the eye (Figure 2 (sections 21–28)). Moreover, *nos1* gene expression occurred in the notochord and in what appeared to be the liver primordium (Figure 2 (section 28)). At stage 28, *nos1* transcript was detected in the embryonic tailbud, and then progressively decreased during development (Figure 2A–C,E,G).

### 3.3. Expression Pattern of nos1 in the Brain of Gar Embryos and Larvae

**Figure 3:**
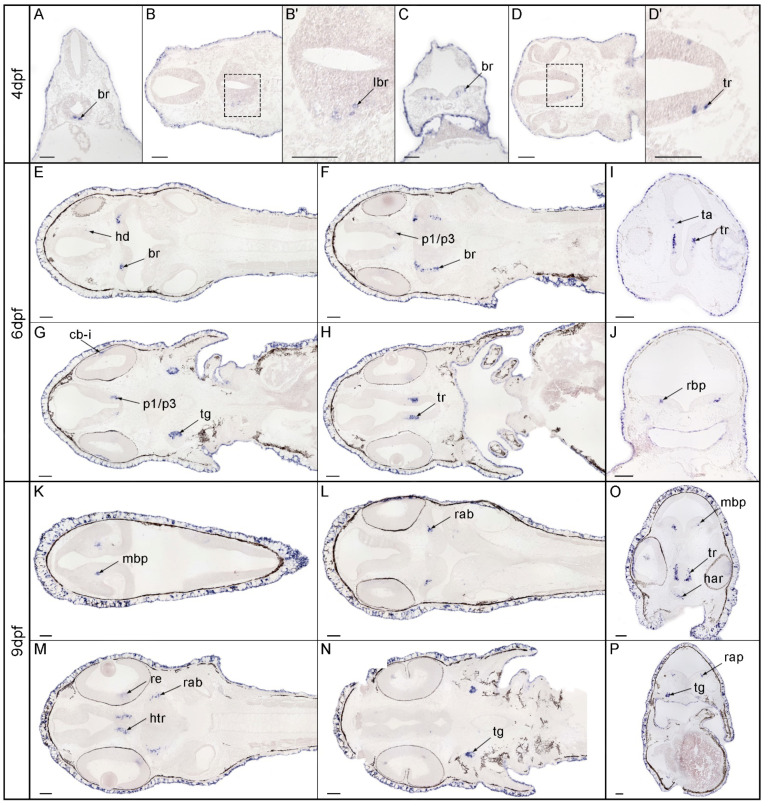
Expression patterns of *nos1* in spotted gar embryos (4-6-9 dpf). At 4 dpf, *nos1*-positive cells were localized in the rhombencephalon (**A**–**D**) in the 4th ventricle (**C**) and in the tuberal/retrotuberal region (**D**,**D’**). At 6 dpf, *nos1* expression localized in the rhombencephalon and the hypothalamic domain (**E**,**F**,**J**), the basal plate of the diencephalon proper p1-p3 (**F**,**G**), the trigeminal ganglia, in the primordium of the ciliary body/iris in the eye (**G**), ventrally in the tuberal/retrotuberal region (**H**,**I**), and the thalamic area (**I**). At 9 dpf, *nos*1 expression was detectable in the basal plate of the mesencephalon (**K**–**O**), the rhombencephalon (**L**,**M**,**O**), the tuberal/retrotuberal region of the hypothalamus and retina (**M**–**O**), and the trigeminal ganglia (**N**–**P**). Abbreviations: br, basal rhombencephalon; cb, ciliary body; har, hypothalamic acroterminal region; hd, hypothalamic domain; i, iris; lbr, latero-basal rhombencephalon; mbp, mesencephalic basal plate; p1/p3, basal plate of prosomere 1 to 3; rab, rhombencephalic alar basal boundary; rbp, rhombencephalic basal plate; re, retina; ta, thalamic area; tr, tuberal region; tg, trigeminal ganglia. Scale bar: 100 µm.

In spotted gar, we analyzed spatial and temporal *nos1* expression patterns at 4, 6, 9, 11, and 14 dpf by in situ hybridization after sectioning in both horizontal and transversal orientations. At 4 dpf (Figure 3A–D), a few *nos1*-positive cells appeared among the postmitotic cells of the basal plate of the rhombencephalon with some of them close to the floor plate (Figure 3A,B,B’); in particular, positive signals became visible in the basal plate of the rhombencephalon in sections through the 4th ventricle (Figure 3C). Some *nos1* positive cells emerged in the tuberal/retrotuberal region of the hypothalamus (Figure 3D,D’). Finally, *nos1* was expressed in the ectoderm associated with the epidermis (Figure 3A–D). At 6 dpf (Figure 3E–J), a positive signal was present in the basal plate of the rhombencephalon (Figure 3E). In more caudal sections, in addition to expression in the rhombencephalon (Figure 3F,G), positive cells were present in the basal plate of the diencephalon proper (p1-p3). The hypothalamic basal plate in the tuberal/retrotuberal region was also *nos1* positive, as in the previous developmental stage (Figure 3H). Moreover, *nos1* was expressed in the trigeminal ganglia and in the eye, both where eye musculature will form and at the level of the primordium of the ciliary body/iris (Figure 3G). Cross sections confirmed *nos1* expression in the tuberal/retrotuberal region and in a few positive cells in the thalamus (Figure 3I) in addition to expression in the basal plate of the rhombencephalon (Figure 3J). Finally, *nos1* continued to be expressed in the ectoderm associated with the epidermis (Figure 3E–H). At 9 dpf (Figure 3K–P), *nos1* expression was detectable in the basal plate of the mesencephalon (Figure 3K) and a positive signal was still present in the rhombencephalon, mainly in the basal plate (Figure 3L,M). Expression was evident in the tuberal/retrotuberal region of the hypothalamus, with a new expression domain in the acroterminal alar plate region, in the retina (Figure 3M,O), and in a position likely to be trigeminal ganglia (Figure 3N). Likewise, cross sections confirmed *nos1* expression in the mesencephalic basal plate, tuberal/retrotuberal region, and the acroterminal alar hypothalamus (Figure 3O). Sections further confirmed *nos1* expression in some derivatives of the basal plate and other domains at the boundary of the basal and alar plates, as well as in trigeminal ganglia (Figure 3P).

**Figure 4:**
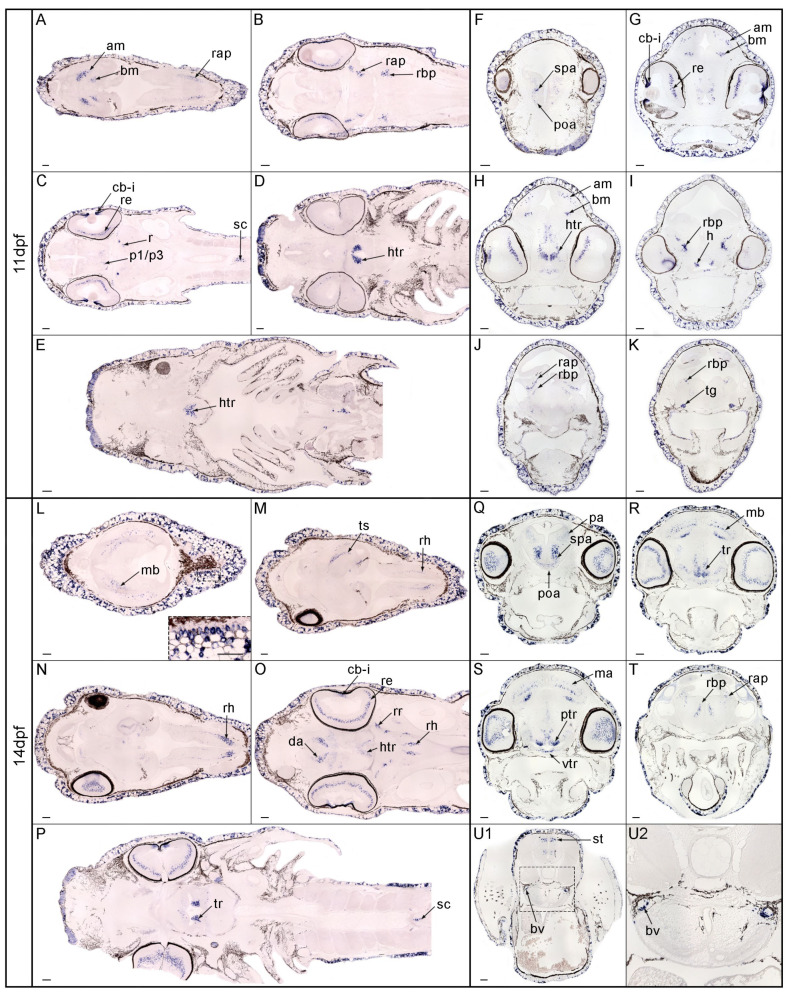
Expression patterns of *nos1* in spotted gar larvae (11–14 dpf). At 11 dpf, *nos1* was expressed in the mesencephalon (**A**), rhombencephalon (**A**,**B**), and eye, spinal cord, rhombencephalon and basal plate of the diencephalic region (**C**). Positive cells were localized in the rhombencephalon (**D**,**I**–**K**) and hypothalamus (**E**–**I**). The expression of *nos1* was detected in the sub-pallial and pre-optic area (**F**), retina, and ciliary body plus iris (**C**–**G**), tuberal/retrotuberal hypothalamus (**H**), mesencephalic area (**G**,**H**) and trigeminal ganglia (**K**). At 14 dpf, positive cells were present in the epidermis (**L**, box), pallium, sub-pallium, and pre-optic area (**L**–**Q**), torus semicircularis (**M**) in the mesencephalic area (**S**), and rhombencephalon (**M**,**N**,**T**). Signal was present in lens and retina (**O**), tuberal region, and spinal cord (**P**,**R**,**S**), nucleus of the solitary tract (**U**), and blood vessel (**U1**,**U2**). Abbreviations: am, alar mesencephalon; bm, basal mesencephalon; bv, blood vessel; da, diencephalic area; htr, hypothalamic tuberal region; ma, mesencephalic area; mb, midbrain; pa, pallium; p1/p3/m, basal plate of prosomere 1 to 3; ptr, periventricular tuberal region; poa, pre-optic area; rap, rhombencephalic alar plate; rbp, rhombencephalic basal plate; rh, rhombomeres; rr, rostral rhombencephalon; re, retina; rh, rhombencephalon; sc, spinal cord; spa, sub-pallial area; st, solitary tract; tg, trigeminal ganglia; tr, tuberal region; ts, torus semicircularis; vtr, ventricular tuberal region. Scale bar: 100 µm.

At 11 dpf (Figure 4A–K), *nos1*-positive cells localized in some derivatives of the alar and basal plate of the mesencephalon (Figure 4A,B), and in the alar and basal part of the rhombencephalon (Figure 4B). At this stage, *nos1* expression in the retina and ciliary body and iris was evident (Figure 4C). As observed in previous stages, high expression was observed in a postmitotic derivative of the tuberal/retrotuberal portion of the hypothalamic region (Figure 4D,E). A new group of *nos1* positive cells was localized in the basal plate of the spinal cord close to the floor plate. This gene was also expressed in some postmitotic derivatives of the basal plate of the diencephalon proper (prosomere 1–3) (Figure 4C). Cross sections confirmed most expression domains. Rostrally, a few cells expressed *nos1* in the sub-pallial and pre-optic area (Figure 4F). In the eye, the retina and primordium of the ciliary body and the iris showed strong signal as well the tuberal/retrotuberal hypothalamus (more evident in Figure 4G–I). Signal was also observed in some derivatives of the alar and basal mesencephalic domains (Figure 4G,H). Positive *nos1* signal was detected in the basal portion of the rhombencephalon (Figure 4I–K). Moreover, signal in the trigeminal ganglia was detected at this developmental stage (Figure 4K). At 14 dpf (Figure 4L–U’), *nos1* expression increased in the integument as well as in several brain areas. Positive cells were present in the epidermis (Figure 4L, box). In the midbrain, *nos1* expression was detected in the optic tectum (Figure 4L) and in the torus semicircularis area (Figure 4M). Caudally, positive cells were localized in the rhombencephalon (Figure 4M,N). The hypothalamic acroterminal and the tuberal/retrotuberal region expressed *nos1* (Figure 4P), as did the diencephalic area. As in previous stages, *nos1* signal occurred in the ciliary body and iris and in a layer across most of the retina (Figure 4O,P). Some cells from the basal plate close to the floor plate of the spinal cord also expressed *nos1* (Figure 4P). Cross sections highlighted signal in the pallium, sub-pallium, and pre-optic area (Figure 4Q). Positive cells were present in the basal plate of the hypothalamic tuberal/retrotuberal region at the periventricular and ventricular strata (Figure 4R). Signal was also observed in the optic tectum and torus semicircularis of the mesencephalic area (Figure 4R,S). Moreover, *nos1* signals were present in the alar and basal plate of the rhombencephalon (Figure 4T) and in its most caudal portion, probably, in the nucleus of the solitary tract (Figure 4U). Finally, a paired signal was evident in an unidentified region of the trunk, possibly blood vessels (Figure 4U1,U2).

## 4. Discussion

Traditionally, studies on the evolution of the central nervous system mainly considered neuroanatomical comparisons of whole brain size or interspecific structural variations. These approaches, however, do not allow a complete understanding of the evolution of neural pathway development. To illuminate evolutionary adaptations that occurred in the nitrergic system of ray-finned fish, we compared *nos1* expression patterns during the development of three actinopterygian species (gar, zebrafish, and medaka), representing key nodes in fish evolution. Ray-finned fish possess a diversified *nos* gene repertoire characterized by several gene duplication and loss events [10].

Comparative analyses among species that diverged millions of years ago is complex due to difficulties in comparing developmental stages between species using character homologies [40]. To make progress, we first employed an empirical staging generalization based on anatomical studies of the three species [30,33,34] because they lack a dedicated comparative characterization at molecular level for specific anatomical and CNS markers. The prosomeric model was used to localize the main common landmarks of the central nervous system during development, main partitions of the brain, and postulate homologies. Following the prosomeric model, the positions of neural populations can be identified by specific gene expression patterns (neural genoarchitecture) [41,42]. Homologues’ derivatives share the same topological position in the Bauplan, an aspect that gene expression patterns help clarify [28,42,43,44,45].

The limited amount of available information, restricted to cyprinids and cichlids, shows that *nos1* starts to be expressed a few hours after fertilization in the developing central and peripheral nervous systems concurrent with the differentiation of embryonic neuronal cell clusters and axonal scaffolds [20,22,23,24,25,46]. Our results obtained from three different fish species confirmed an increase of *nos1* mRNA-expressing cells in some territories in common among all three species, but also in species-specific expression domains (Table 1).

Expression of *nos1* in the telencephalon was detected only in zebrafish at early stages and at later stages of development in all three selected species. In contrast, diencephalic expression of *nos1* was detected early for all fish, but only gar and medaka maintained expression in the diencephalon throughout development. In the mesencephalon, *nos1* expression appeared in the embryo only in zebrafish and gar, but in larval stages, the mesencephalon showed similar expression patterns in all three fish species. In the rhombencephalon, *nos1* expression persisted from the late embryo stage throughout development in all three species.

In general, our results showed common *nos1* expression patterns in brain territories of the three species tested here, suggesting that the gar expression could be highly similar to the common ancestral state and that this pattern has also been maintained in zebrafish and medaka.

While the CNS in the three investigated species had many common expression areas, other structures and peripheral organs provided different outcomes. The *nos1* expression pattern in the developing eye has been described in teleosts [24,25,47] and is confirmed in our study. In gar, *nos1* expression in the eye appeared at an earlier developmental stage than in the two teleosts, allowing one to speculate either that *nos1* has roles in eye formation in gar missing from teleosts or that it has the same role in gar and teleosts but that the eye is developing somewhat more rapidly in gar than in the teleosts relative to other organs. Interestingly, *nos1* positive cells appeared in the primordia of the thyroid exclusively in medaka, and liver in medaka and in zebrafish as shown by hybridization experiments (this work) and scRNA-seq data [39], respectively. In fish, a crosstalk between thyroid hormones and nitric oxide signaling pathways was demonstrated [48], although a direct role of NO has not yet been described in thyroid development. Analogously, the expression of *nos* genes in adult teleost liver was recently reported (reviewed in [49]), but the functions of *nos1* in liver development remain to be established. These findings raise questions about specific roles NO might play in the growth, specialization, and physiology of these organs.

A peculiar signal was found in the tailbud of medaka early embryos, as previously reported [22], and here we also observed *nos1* expression at later stages in this structure. In fishes, a pool of large neurosecretory neurons is located in the terminal segments of the spinal cord: the caudal neurosecretory system (CNSS) [50]. In this structure, NO acts as a local synchronizer for the neighboring neurosecretory cells [51]. The onset of the CNSS starts during early development stages, as demonstrated for Nile tilapia, *Oreochromis niloticus* [52], and chum salmon, *Oncorhyncus keta* [53]. Therefore, the presence and distribution of neuronal *nos1* in medaka tailbud could be related to this neurosecretory system, but further experimental evidence is needed to test this hypothesis. Remarkably, NO synthesis was also reported in the caudal portion of the hindgut and anal region in the cephalochordate *Branchiostoma lanceolatum* [54]. It would be interesting to investigate the putative conservation of CNSS as well as physiological pathways in basally diverging chordates. The presence of *nos1* expression in the fish skin has been associated with several roles, including the regulation of epithelial secretory activities linked to mucosal immunity [55]. Transient expression in epithelial cells was reported in zebrafish from 20 hpf to hatching stage [25] and our experiments support the putative involvement of NO in the skin both in zebrafish and gar, highlighting a strong gene expression in peridermal cells in all analyzed developmental stages.

In conclusion, the ancient and fundamental Nos1 enzyme, highly structurally conserved over evolution, has maintained its main expression domains in the CNS in teleosts and their sister group the holosteans, and so these domains likely represent the ancestral condition. On the other hand, diversified *nos1* expressions in liver, thyroid gland, peripheral nervous system, and caudal neurosecretory system prompt the hypothesis that they could be linked to species-specific neurogenetic and developmental processes, which requires future investigation.

## Figures and Tables

**Table 1:** Comparative analysis of *nos1* expression territories in zebrafish, medaka, and spotted gar.

	Species (Stage)	Head Region						Trunk Region					
		Tel	Di	Mes	Rho	Eye	Cn	Liv	Th	PNS	Nt	Sk	Tb
Early Embryo	Zebrafish (24 hpf)	+	++									++	
	Medaka (st 28)		+				+++		++				+++
	Gar (4 dpf)		+		+							+++	
Late Embryo	Zebrafish (48 hpf)	++		++	++								
	Medaka (st 30)				++		++		++				++
	Gar (6 dpf)		++		++	+	++					+++	
Early Larva	Zebrafish (72 hpf)	++		++	++								
	Medaka (st 32)	+++			+		++		+	++	+		++
	Gar (9 dpf)		+	+	+	+	+					+++	
Late Larva	Zebrafish (96 hpf)	++		++	++	++	++						
	Medaka (st 34)	++	++	++	+++	++	++	+++			++		++
	Gar (11–14 dpf)	+++	+++	++	+++	+++				++	++	+++	

Staging in zebrafish according to Kimmel et al. 1995 (hpf), in gar, to Long and Ballard 2001 (dpf) and in medaka, to Iwamatsu 2004 (stage). Abbreviations: Cn, Cranial nerves; Di, Diencephalon; Liv, Liver; Mes, Mesencephalon; Nt, Notochord; PNS, Peripheral Nervous System; Rho, Rhombencephalon; Sk, Skin; Tb, Tailbud Tel, Telencephalon; Th, Thyroid. Visual scoring, + indicates the measure of nos1-positive cells: + low, ++ average, +++ high.

## Data Availability

Not applicable.

## Supplementary Materials

The following supporting information can be downloaded at: www.mdpi.com/article/10.3390/genes13050918/s1, Table S1: Primers used to synthetize riboprobes. Figure S1: zebrafish scRNA-seq data analysis.
