# Supplementary material for: Expression Pattern of nos1 in the Developing Nervous System of Ray-Finned Fish
Source: Genes (Basel). 2022 May 20;13(5):918. doi: 10.3390/genes13050918 (PMC9140475; doi:10.3390/genes13050918)
Supplement: Supplementary file 1 [file genes-13-00918-s001.zip › genes-1694900-supplementary.pdf]

Supplementary Table S1

Primers used to synthesize riboprobe for *in situ* hybridization.			
**Species**	**Acc. Number**	**Sequence (5' > 3')**	**Riboprobe size**
Danio rerio	NM_131660	*Fw* - CAAGCAGCGGGTGTGCAAAC	908 bp
		Rv - CTATGCAAACGTGCTCAGTG	
Oryzias latipes	NM_001104855	*Fw* - GTATATGGGCACAGAGATCG	953 bp
		Rv - CTTCAAACAGCGTGTCCAC	
Lepisosteus oculatus	XM_015366414	*Fw* - CGACATGAGCTATGAGAGCG	943 bp
		Rv – GTCCACATGTGCCTTAGAGC	

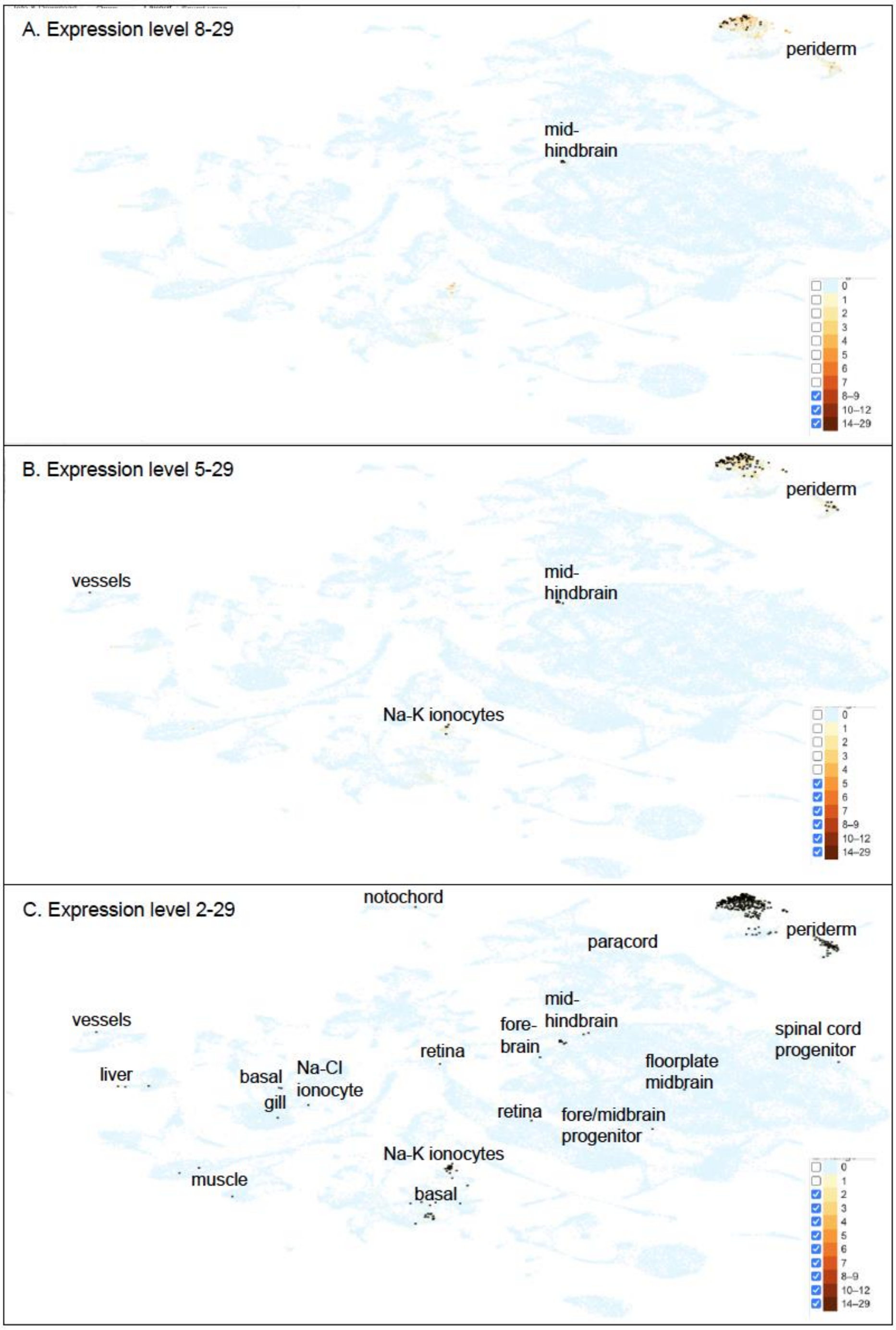

Supplemental Figure S1. *nos1* expression in scRNA-seq data.
A. At the highest levels of expression (levels 8 to 29), *nos1* was expressed in 30 cells that include periderm cells and a highly specific group of midbrain-hindbrain neurons.
B. At high and midlevel expression (levels 5 to 29), *nos1* was expressed in 112 cells that included the more periderm and midbrain-hindbrain cells, and in addition, several Na-K ionocytes, and strongly in one blood vessel cell.
C. At high, mid-, and low levels (levels 2 to 29), *nos1* transcript was present in additional cells, including muscle, basal skin cells, gill, retina, and in the CNS in the forebrain, floorplate of the midbrain, and in spinal cord progenitor cells.

Data were extracted from the zebrafish gene expression atlas (Farnsworth *et al.*, 2019), and include embryos at 1 and 2 dpf and larvae at 5 dpf. Color scale for expression levels shown at lower right of each panel. Cell identities determined as in Farnsworth *et al.* (2019).

Farnsworth DR, Saunders LM, Miller AC. A single-cell transcriptome atlas for zebrafish development. Dev. Biol. (2020) 459: 100-108. doi: 10.1016/j.ydbio.2019.11.008.
